# Community-based clinic volunteering: an evaluation of the direct and indirect effects on the experience of health science college students

**DOI:** 10.1186/s12909-016-0547-y

**Published:** 2016-01-18

**Authors:** Yelena Bird, Adiba Islam, John Moraros

**Affiliations:** School of Public Health, University of Saskatchewan, 104 Clinic Place E-Wing Health Sciences, Room 3322, Saskatoon, SK S7N 5E5 Canada

**Keywords:** Volunteerism, Evaluation, Satisfaction, Performance, Overall experience, College students, Community-based clinic

## Abstract

**Background:**

The present study was conducted in a multi service-learning, student managed and operated, community-based clinic. Its aim was to measure the direct and indirect effects of how proximal factors (i.e., ‘management’, ‘support received’, ‘duration of involvement’, and ‘average time spent per month’) and mediators (i.e., ‘training received’, ‘motivation’, and ‘commitment’) influence distal outcomes (i.e., ‘performance’, ‘satisfaction’, and ‘overall experience’) within a volunteer organization.

**Methods:**

Participants were recruited through the use of an email list server. An online survey was used containing multi-item measures from validated scales. Data were collected from 170 volunteers from July to August 2013. Data analysis used a structural equation modeling (SEM) framework for the estimation of direct and indirect effects on constructs and variables of interest. Only statistically significant relationships were reported at *p* < 0.05.

**Results:**

In this study, there are several direct effects worthy of note. First, the proximal factor of ‘management’ plays an important role in influencing the mediators of ‘motivation’ (standardized beta = 0.55) and ‘training received’ (0.65) by the student volunteers but has a relatively small impact on their ‘commitment’ (0.39) to the organization. Second, the mediator of ‘motivation’ proved to have the strongest impact on the distal outcome of volunteer ‘performance’ and ‘satisfaction’ levels (0.41 and 0.58 respectively), whereas ‘commitment’ (0.44) was the key in determining their ‘overall experience’ with the organization. These results in turn, help contextualize the indirect effects observed in our study. Namely, the proximal factor of ‘management’ played a distinctive role in influencing the distal outcomes of volunteer ‘performance’ (0.32) and ‘overall experience’ (0.66), whereas the organizational ‘support received’ by the volunteers was key to their ‘satisfaction’ (0.21).

**Conclusions:**

The findings of the present study shed light into the direct and indirect effects of how proximal factors and mediators, influence student volunteers distal outcomes within a community-based clinic. These results provide useful information and serve as a valuable tool to higher education (curriculum experts, accreditation specialists, students, faculty and administrators) and non-profit community organizations (clients, staff and managers) in their efforts to improve student volunteer satisfaction and performance outcomes.

## Background

Voluntarism is defined as the practice of freely and without compensation giving one's time or talents for charitable, educational, or other worthwhile activities for the purpose of benefitting others, especially in one's community [1]. In Canada, nearly one-half of its population aged 15 years old and older (about 13 million people) performed volunteer work in 2010. These Canadians devoted almost 2.07 billion hours to their volunteer activities, which equates to approximately 1.1 million full-time jobs [2].

While the rates of volunteering across Canada vary considerably, the highest rate was recorded in the province of Saskatchewan, where it was estimated that 58 % of people aged 15 years old and older did volunteer work in 2010. Among Saskatchewan volunteers, young people, predominantly students aged 15 to 24 years old, reported the highest rates at 66 % [2]. Not surprisingly, civic engagement and volunteer community service initiatives on Canadian college campuses in general and the University of Saskatchewan specifically are significant in scope and growing in number.

Previous studies have shown that college-aged students volunteer for different motives and perceived benefits than other people. A major motivator for young people and college students in particular is the opportunity to gain work-related experiences, develop skills, and build on qualifications that can tangibly help them attain their educational goals and further their professional careers [3]. Therefore, career related benefits usually dominate the volunteering discourse as college students recognize the need to build their resumes [4] and personal capital [5]. This has led to the establishment of a plethora of volunteer activities across university campuses in Canada.

One such major volunteer initiative is a student managed and operated multi service-learning, community-based organization that provides much needed healthcare services to the poor and underserved core neighborhoods of Saskatoon, Saskatchewan. It was founded in 2005 by a handful of pioneering University of Saskatchewan medical students. Since that time, student volunteers from across health science disciplines and other Saskatchewan educational institutions have joined to make significant contributions to the project [6].

This organization is governed by a Board of Directors or “Council”, which is comprised of nine student volunteer members with voting privileges and four advising but non-voting members (two professional volunteers, one volunteer from the community, and the executive director). It is administered and run primarily by students in the health sciences at all levels of training and supervised by practising physicians and community professionals [6]. Administrative supports are provided by a handful of dedicated staff members and the executive director.

Recruitment of student volunteers by the organization usually takes place at the beginning of each academic year (late August to early September) but students have the opportunity to join at any time. Volunteers are recruited by word of mouth and through university-wide emails. There are a number of health science student organizations at the University of Saskatchewan campus and especially the medical school student organization, which do an excellent job recruiting students to volunteer. Interested students sign up for email notifications, complete the necessary volunteer paperwork, go through a background check, enter an orientation program, and start attending volunteer activities and organizational meetings.

Once officially approved and satisfactorily trained, student volunteers get assigned into interdisciplinary, primary care teams, which typically see patients. These teams usually consist of a good mixture between underclassman (first and second year) and upperclassman (third and fourth year) health science (mostly medical) college students. Each team is responsible for taking the patient’s medical history, conducting an interview specific to the cause of the patient’s medical visit, and completing a physical exam if indicated. The team then presents the patient to the attending physician/mentor. The case is discussed, and the team along with the attending physician see the patient to conclude the visit. When appropriate, the patient is referred to outside healthcare services or to in-house patient care, education, nutritional support (including meals), and child care services. Each team is responsible for documenting the patient encounter in the clinic’s electronic medical records. The documentation is prepared and submitted by the interdisciplinary, primary care team and then it gets reviewed and signed by the attending physician/mentor.

Despite increased interest in similar student-led volunteer initiatives, a thorough review of the literature revealed there have been few studies published, in this type of setting [7–10]. The most comprehensive survey on the topic was conducted by Simpson and Long, who concluded that student-run health clinics have not been well studied and existing data is limited upon which to base future investigation of these programs [11]. To the best of the authors knowledge, there is currently no published research examining the specific constructs (i.e., management, motivation, commitment, performance) used in this study in order to identify how they are inter-related and measure their direct and indirect influence on volunteer experience within a student-run clinic.

Therefore, the present study helps fill a basic gap in our knowledge on this important topic. It uses the broader framework of the exchange theory [12] to help establish our model constructs (i.e., proximal factors, mediators, and distal outcomes) and explain our findings. This theory posits that the social behavior of individuals (in this case, college student volunteers) is the result of an exchange process. The purpose of the exchange process is to maximize benefits and minimize costs. Within this context, the aim of the study was to measure how proximal factors (i.e., ‘management’, ‘support received’, ‘duration of involvement’, and ‘average time spent per month’) and mediators (i.e., ‘training received’, ‘motivation’, and ‘commitment’) influence distal outcomes (i.e., ‘performance’, ‘satisfaction’, and ‘overall experience’) for student volunteers within a unique multi service-learning, student managed and operated, community-based clinic.

## Methods

### Data collection and instruments

In this study, a survey was used containing multi-item measures from validated scales [13, 14]. Data were collected between July and August, 2013. Volunteers were invited to participate in the study via email and represented both current and former members of the community organization.

Overall, the survey consisted of 31 close-ended questions but participants were also provided with an opportunity to offer any additional comments in an open-ended section at the end of the survey. The survey contained questions that asked about the student volunteer’s socio-demographic characteristics, affiliation with the community organization, motivation, commitment, performance, satisfaction, and overall experience. Participation in the study was voluntary with no tangible incentives provided to the student volunteers. Consent to participate was implied by completion of the survey. The Behavioral Research Ethics Board at the University of Saskatchewan approved this study (BEH#13-201).

The Qualtrics software platform was used to distribute the survey online to study participants. This ensured that participant anonymity was maintained throughout the data collection process. Best-practice guidelines were used in our survey design and delivery [15]. In the survey, only one question was presented per screen so at to make it easier to scroll down to the next question. Additionally, progression to the next question was not allowed until the respondent completed the previous question. These measures helped ensure a higher question completion rate. An electronic reminder was sent out each week to all potential study participants. On the last week, two reminders were sent out. Survey statistics on the Qualtrics platform showed that response rates in this study were often 10–20 % higher on the days that reminders were emailed. The total response rate was approximately 34 %.

### Participants

The socio-demographic characteristics of the study participants are presented in Table 1. In brief, there were a total of 170 student volunteers, who responded to the survey and completed at least 70 % of the questions. In this group, there were two and a half times more females (72 %) than males (28 %). A large majority were between the ages of 18 to 24 years old (68 %). Furthermore and considering their age group, it is not surprising that they were single (87 %) and had no children (95 %). Additionally, nearly half (47 %) reported an annual individual income (after taxes) that was less than $6000 CAD. These characteristics are consistent with the fact that most study participants were college aged students in the midst of their post-secondary education, while volunteering at the community organization.

**Table 1:** Socio-demographic characteristics of student volunteers

Socio-demographic characteristics	Frequency	Percent (%)
Sex		
Male	48	28.2
Female	122	71.8
Age group		
18–24 years	116	68.2
25–31 years	45	26.5
32 years or more	9	5.3
Marital Status		
Single	148	87.1
Married	14	8.2
Common-Law	8	4.7
Children		
Yes	9	5.3
No	161	94.7
Employment status		
Unemployed	54	31.8
One job	82	48.2
Two jobs	21	12.4
Other	13	7.6
Annual individual income (after taxes):		
Less than $6000	80	47.1
$6000-$10,999	45	26.5
$11,000-$15,999	16	9.4
$16,000-$20,999	9	5.3
$21,000 or more	20	11.8
Highest level of education completed		
Less than high school	2	1.2
High school diploma	59	34.7
College or trade certification	3	1.8
Undergraduate university degree	91	53.5
Graduate university degree	15	8.8
Total	170	100.0

### Structural equation modeling

To better understand the interrelationship between the various constructs used in this study and simultaneously evaluate their indirect and direct effects, a model was built using path analysis on the structural equation modeling (SEM) software MPlus [16, 17]. Path analysis was chosen because it is equivalent to running simultaneous regression models within the SEM framework. In brief, the model consists of three variable categories and 10 constructs: 1) Proximal Factors (i.e., ‘management’, ‘support received’, ‘time spent per month’, and ‘duration of involvement’), 2) Mediators (i.e., ‘motivation level’, ‘commitment level’, and ‘training received’) and 3) Distal Outcomes (‘performance level’, ‘satisfaction level’, and ‘overall experience’). All the constructs exhibited high reliabilities (i.e., minimal measurement errors), which permitted averages to be calculated to fit the structural model. This modeling framework allowed for estimation of both direct and indirect (i.e., mediating) effects in addition to regression coefficients for each relationship between key constructs. Statistically significant relationships are reported by comparing standard deviations (SD) at *p* < 0.05.

## Results

### Volunteers’ degree of affiliation with the community organization

Different aspects of the student volunteers’ affiliation to the community organization were surveyed in this study, as shown in Table 2. While there was a relatively even distribution with regard to the length of the time period they were involved with the community organization (<3 months: 22 %; 3–6 months: 21 %; 7–9 months: 14 %; 10–12 months: 13 %; > 12 months: 31 %), the majority (72 %) volunteered for less than 10 h per month. Many of the volunteers first became aware and chose to get involved with the community organization after hearing about it from their academic institution (58 %) or from people they knew, such as their friends (37 %). Very few volunteers (1 %) joined the organization as a consequence of being exposed to promotional materials (i.e., website, brochures/posters). The initial reasons for joining the organization were largely due to their interest to gain new experiences (39 %), to give back to the community (22 %), and to help vulnerable groups (18 %). Some volunteers also indicated that their initial motive was to strengthen their resume (14 %).

**Table 2:** Affiliation with the community organization

Affiliation with the community organization	Frequency	Percent (%)
Duration of involvement		
Less than 3 months	37	21.8
3–6 months	35	20.6
7–9 months	23	13.5
10–12 months	22	12.9
Greater than 12 months	53	31.2
Time, on average, contributed per month		
Less than 10 h	123	72.4
10–19 h	36	21.2
20 h or more	11	6.5
How did you first hear about this community organization?		
From people I know (i.e., friends)	63	37.1
Online (i.e., website)	4	2.4
At an academic institution	99	58.2
At another community organization	2	1.2
At my workplace	1	0.6
Brochures/posters	1	0.6
What was the underlying reason why you originally chose to volunteer here?		
To broaden your social network	2	1.2
To help vulnerable groups	30	17.6
To gain new experiences	67	39.4
To give back to the community	37	21.8
To strengthen your resume	24	14.1
Other	10	5.9
Has your original perspective changed with respect to why you volunteer here?		
Yes	82	48.2
No	88	51.8
If “yes”, what is the current underlying reason why you continue to work here?		
To broaden your social network	3	3.7
To help vulnerable groups	26	31.7
To gain new experiences	18	22.0
To give back to the community	22	26.8
To strengthen your resume	2	2.4
Other	11	13.4
Did you have prior volunteer or work experience(s) prior to joining here?		
Yes	142	83.5
No	28	16.5
After joining this community organization, are you volunteering or working anywhere else?		
Yes	68	40.0
No	102	60.0
Have you recommended others (i.e., friends, family) to volunteer or work here?		
Yes	145	85.3
No	25	14.7

### Volunteers’ evaluation of the community organization and their own experience

The student volunteers’ evaluation of the organization and their own experience are presented in Table 3. It details the mean and standard deviation for the questions pertaining to the proximal factors (i.e., ‘management’, ‘support received’, ‘duration of involvement’, and ‘average time spent per month’), mediators (i.e., ‘training received’, ‘motivation’, and ‘commitment’) and distal outcomes (i.e., ‘performance’, ‘satisfaction’, and ‘overall experience’). In general, the feedback was positive as mean values for the student volunteer ratings of the organization often ranged between the 70^th^ and 80^th^ percentile.

**Table 3:** Measuring the key constructs of a structural model used to evaluate the experience of student volunteers in a community-based organization (*N* = 170)

CONSTRUCTS	MEAN	SD
PROXIMAL FACTORS		
Management		
I am fully aware of my roles and responsibilities.	77.68	20.91
I am given a sufficient amount of time to fulfill my duties.	84.88	17.30
I have the resources and facilities to complete my assigned tasks properly.	83.29	18.39
I receive appropriate and timely feedback for the tasks I perform.	71.53	23.08
Support received		
My opinions are listened to.	76.35	22.41
My achievements and contributions are recognized.	74.50	24.21
My interests are taken into consideration for the duties I am asked to perform.	76.47	23.78
My concerns and inquiries are addressed.	79.56	21.95
MEDIATORS		
Motivation level		
When I perform well, I know it’s because of my own desire to achieve.	81.85	18.40
Becoming an integral member of the team is something I want to do for myself.	77.65	21.42
If I were independently wealthy, I would still volunteer here.	84.29	20.15
I feel that I am performing a useful and very much needed service.	80.06	22.49
I do not mind at all that I am not getting paid for my participation.	92.71	13.18
Commitment level		
I am extremely loyal.	67.26	25.44
This is my first choice for a community organization to work at.	70.24	26.66
I am very knowledgeable about this organization (i.e., programs and services).	67.32	22.99
When I work here, I feel important.	67.79	25.93
My preference for this organization as an ideal workplace would not willingly change.	68.56	23.35
I care about the long-term success of this place and its influence in the community.	87.15	18.92
Training received		
I received training for my specific roles and responsibilities.	69.85	22.61
I gained knowledge on the organization’s mission, main goals, and objectives.	81.59	19.42
The training sessions had informative and practical knowledge.	73.12	23.33
I applied the knowledge I acquired from the training sessions to my duties.	72.71	24.23
DISTAL OUTCOMES		
Performance level		
I complete my duties in a timely manner.	84.94	14.31
I achieve beyond what is expected of me on a regular basis.	72.29	18.34
I show improvement in the tasks I perform.	78.35	15.44
Client satisfaction is my top priority.	85.74	13.76
I am able to communicate cordially with my fellow colleagues.	87.00	14.09
I am good at my job.	80.53	14.13
Satisfaction level		
My work is like a hobby to me.	71.79	23.21
I am always enthusiastic about my work.	77.11	19.09
My job is usually interesting enough to keep me from getting bored.	75.77	22.28
I enjoy my work more than my leisure time.	53.36	26.72
I feel that I am happier in my work than most other people.	68.60	22.69
I feel a sense of pride and accomplishment from the work I do.	81.79	18.71
Overall experience		
The work I do is meaningful.	79.49	19.58
The work I do is challenging.	64.23	26.82
My work offers me a career path that I am pleased with.	73.18	23.37
I have built long-lasting relationships as a result of my work.	62.83	26.36
I am able to balance my personal and professional life.	73.42	21.73
I like the work I do.	78.24	21.93

#### Structural model analysis –direct effects

**Fig. 1:**
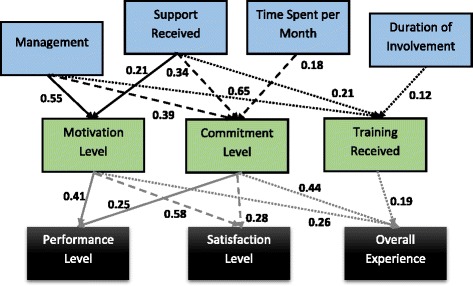
Structural model analysis–direct effects

The structural model in Fig. 1 was used to estimate the direct effects in our study between proximal factors, mediators and distal outcomes. The values represent standardized coefficients, which allow us to see the relative importance of these direct effects. For example, among the proximal factors, ‘management’ has the greatest positive influence on mediators such as a volunteer’s ‘motivation’ (standardized beta = 0.55), ‘commitment’ (0.39), and ‘training received’ (0.65). With respect to the distal outcomes of a volunteer’s ‘performance’ and ‘satisfaction’, the mediating effect of ‘motivation’ has the strongest impact (0.41 and 0.58, respectively). By comparison, when examining the distal outcome of ‘overall experience’ for the volunteers, the mediating effect of their ‘commitment’ to the organization appears to be the key player (0.44).

#### Structural model analysis – indirect or mediator effects

**Fig. 2:**
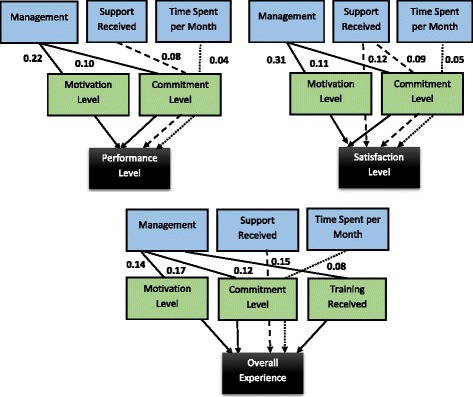
Structural model analysis–indirect or mediator effects

The next structural model analysis used in this study, examined the question of how the distal outcomes of ‘performance level’, ‘satisfaction level’, and ‘overall experience’ are indirectly effected by proximal factors such as ‘management’, ‘support received’, ‘time spent per month’, and ‘duration of involvement’. The indirect or mediator effect was examined by taking into account the role played by the volunteer’s ‘motivation level’, ‘commitment level’, and ‘training received’. This analysis is presented in Fig. 2 and it is important as it provides keen insight and helps to further our understanding of volunteer behaviour at this community organization.

The model displays both the specific and the total indirect effects. For example, the specific indirect effect of ‘management’ on ‘motivation level’ is 0.22 (i.e., 0.55 × 0.41, as shown by the standardized coefficients in Fig. 1). By comparison, the specific indirect effect of ‘management’ on ‘commitment level’ is 0.10 (i.e., 0.39 × 0.25 from Fig. 1). Hence, the total indirect effect of ‘management’ on volunteer ‘performance level’ through these two mediators (i.e., ‘motivation’ and ‘commitment level’) is 0.32 (i.e., 0.22 + 0.10).

In Fig. 2, there are a number of statistically significant relationships. Of note, ‘management’ played a key role with regard to a volunteer’s ‘performance level’, ‘satisfaction level’, and ‘overall experience’ through its influence on their ‘motivation level’, ‘commitment level’, and ‘training received’. For example, ‘management’ as a proximal factor was influenced by the mediating effects of a volunteer’s ‘motivation’ and ‘commitment level’ leading to increases in their distal outcomes of ‘performance level’ (0.22 and 0.10), ‘satisfaction level’ (0.31 and 0.11) and ‘overall experience’ (0.14 and 0.17 respectively) with the organization.

On the other hand, the proximal factor of ‘support received’ played its most important role in determining a volunteer’s ‘satisfaction level’ (a distal outcome) by acting through the mediators of ‘motivation’ and ‘commitment level’ (increases by 0.12 and 0.09, respectively). By comparison, the proximal factor of ‘time spent per month’ (less than versus more than 10 h) had minimal effect on the distal outcomes of volunteer ‘performance level’, ‘satisfaction level’, and ‘overall experience’.

Finally, while a volunteer’s ‘duration of involvement’ with the organization was included in the combined regression model, it did not have a significant relationship at *p* < 0.05 and therefore, it is not shown in Fig. 2. However, it is worthy to note that the influence of ‘duration of involvement’ did have a *p*-value less than 0.10 as did the majority of indirect effects not displayed in Fig. 2. This indicates that with a larger sample size, it is possible that many of the indirect effects of the Proximal Factors (‘management’, ‘support’, ‘time spent per month’, and ‘duration of involvement’) could be statistically significant.

## Discussion

The results of the present study can be better understood within the broader framework of the exchange theory that looks to maximize an individual’s benefits and minimize their costs [12]. Volunteering aligns with this theory as it provides college students with opportunities to learn new things, built their social networks, strengthen their resumes and enhance their self-confidence through an exchange relationship [18]. Notably, a number of the students reported a change in their perspective as to why they volunteered their time with the community organization. It appears that their volunteer experience had meaningfully reshaped and transformed their initial perspective from one of ‘resume building’ and ‘skill development’ to one based in a desire to ‘help vulnerable groups’ and ‘give back to the community’. This finding is not unique to our study but rather consistent with and widely supported by the existing literature [19–21].

In regard to the ‘training received’, the student volunteers felt that they were well informed about the organization’s mission, main goals, and objectives but wanted to receive more training specific to their roles and responsibilities. In terms of ‘management’, they believed they were given sufficient time to fulfill their duties and had the resources and facilities necessary to complete their assigned tasks properly but felt receiving more timely feedback on the tasks they performed was an area that needed some improvement. Insofar as the ‘support they received’, volunteers felt their opinions were respected, contributions acknowledged, interests considered, and concerns addressed by the organization. This in turn, may help explain their documented high ‘motivation level’ with regard to volunteering at the community organization.

However, while their ‘motivation’ was high, their ‘commitment level’ to the organization was lower by comparison (e.g., most of them only spend less than 10 h/month). Thus, even though the volunteers felt like they were performing a worthwhile and useful service, they did not seem to be fully committed to the organization. Paradoxically, they reported not having a strong attachment to the organization even though they expressed to care about its long-term success and influence in the community.

With respect to their ‘satisfaction level’, volunteers felt a sense of pride and accomplishment from their work but did not feel that volunteering at the organization was more enjoyable than their leisure time. Yet, despite their relatively lower ‘commitment’ and ‘satisfaction levels’, they rated their ‘performance’ high. They felt they performed their duties in a timely manner and on a regular basis. They thought they were friendly with colleagues and good at putting the clients first. Lastly, in terms of their ‘overall experience’, they believed their time at the organization was meaningful and relatively well-aligned with their career paths but felt it could be made more challenging and lacked opportunities to build long-lasting relationships.

The findings from the structural model analysis of our study make it clear that there are several constructs with direct effects worthy of note. First, ‘management’ plays a key role in influencing ‘motivation’ and the ‘training received’ by the student volunteers but has a relatively small impact on their ‘commitment’ to the organization. Second, with respect to volunteer ‘performance’ and ‘satisfaction’ levels, ‘motivation’ has the stronger impact, whereas for ‘overall experience’, it is evident that ‘commitment’ is the bigger player.

These results in turn, help contextualize the indirect effects observed among the constructs in our study. Namely, ‘management’ played a distinctive role in influencing volunteer ‘performance’, ‘satisfaction’, and ‘overall experience’ through the use of mediators such as ‘motivation’, ‘commitment’, and ‘training received’. Additionally, the organizational ‘support received’ by volunteers played a key role with respect to their ‘satisfaction’ by acting through the mediators of ‘motivation’ and ‘commitment’.

The results of the current study add to the body of knowledge on student volunteerism. By focusing on social exchanges, it identifies aspects that may be useful and beneficial to academic institutions and volunteer organizations. Specifically, it was shown that ‘management’ and the ‘support received’ by student volunteers (proximal factors) were critical in influencing their ‘motivation’ and ‘commitment’ (mediators) leading to an increase in their ‘performance’, ‘satisfaction’ and ‘overall experience’ (distal outcomes) with the organization. Consistent with previous research, the present study also showed evidence that overall, student volunteers felt like they were performing a worthwhile and useful service that was meaningful to the organization, well-aligned with their career paths, and of benefit to their community [20–23].

### Strengths and limitations

The current study has several strengths. Previous research has shown volunteerism to be a beneficial experience to students and our study supports these findings [24, 25]. In recent years, college students especially those who study in health science disciplines have demonstrated an increased interest and active involvement in volunteerism [26–28]. This creates the critical mass necessary to effect change through capacity building. The results of our study help identify the various factors that determine a student volunteer’s motivation and commitment to civic involvement. Specifically, it measures how motivation and commitment, in turn, influence the student volunteer’s performance, satisfaction, and overall experience within a student run, community-based clinic.

College students from the health sciences can be a valuable resource to many community-based healthcare organizations that may be lacking the manpower or funding to support the hiring of full-time staff to serve the mission of the organization. Financial constraints in healthcare in general and non-profit community organizations in particular have increased the importance of the role that student volunteers can play. Conversely, student volunteers rely on these community organizations to provide them with significant opportunities for learning and service.

This study also has a number of limitations. First, the study design is cross-sectional and therefore, it can imply association but not causation. Second, the data are self-reported and may be subject to a response bias to the extent that volunteers with more positive experiences were potentially more likely to respond to the survey. Third, it is unclear whether a greater number of former or current student volunteers responded to the survey and as the management of the organization changes every few years, this could influence their experience with the organization. Finally, the findings of our study while applicable to student volunteers at the specific community organization may not be generalizable to other populations and settings (i.e., non-health science students and organizations that are not student-run).

### Implications for practice

This study provides a roadmap to better understand how motivation and commitment, in turn, influence the student volunteer’s performance, satisfaction, and overall experience. Additionally, it helps to identify specific issues such as the need to offer volunteers more training specific to their roles and responsibilities, provide more timely feedback on the tasks performed, make the experience more challenging and facilitate opportunities to build long-lasting relationships as areas that require improvement. These findings have significant implications for institutions of higher learning and community-based organizations. Such knowledge can be used in the design and implementation of strategies to ensure a positive experience for student volunteers leading to higher commitment and satisfaction levels.

### Recommendations for future research

There are a number of scholarly avenues for future researchers to explore in this exciting and growing field. Here, we provide but a few. Future research can use a longitudinal study design in order to track outcomes on the long-term effectiveness of the student-run clinic experience in affecting health science students practice, behaviors and attitudes in healthcare settings. Another area to consider is to investigate the possible comparisons and differences between first-time as opposed to returning/continuing student volunteers. Finally, in addition to quantitative research, qualitative research employing focus group, in-depth key informant interviews as well as field observations can be used to provide rich text analysis and help deepen our understanding on how to best address possible gaps in the literature with regard to student volunteers’ motivation, commitment and satisfaction.

## Conclusion

The findings of the present study shed light into the direct and indirect effects of how proximal factors and mediators, influence distal outcomes for student volunteers within a community-based clinic. These results can provide useful insight and serve as a valuable tool to higher education (curriculum experts, accreditation specialists, students, faculty and administrators) and non-profit community organizations (clients, staff and managers). It can help inform the planning and evaluation of effective programs that will provide an improved performance and satisfaction experience to the student volunteers while at the same time accomplish the community organization’s goals and objectives. However, additional research is needed in this area, especially within the context of a longitudinal study of a community-based organization that is managed and operated by the student volunteers themselves. This unique demographic and setting, hold great promise and untapped potential for meaningful volunteer work in the future.

## Abbreviation

SEM: structural equation modeling
