# Correction to: Flipping for success: evaluating the effectiveness of a novel teaching approach in a graduate level setting

**DOI:** 10.1186/s12909-017-1027-8

**Published:** 2017-11-10

**Authors:** John Moraros, Adiba Ashrafi, Stan Yu, Ryan Banow, Barbara Schindelka

**Affiliations:** 1 School of Public Health, University of Saskatchewan, 104 Clinic Place, E-Wing Health Sciences, Room 3320, Saskatoon, SK S7N 2Z4 Canada; 2 Research and Program Evaluation Analyst, Gwenna Moss Centre for Teaching Effectiveness, University of Saskatchewan, Saskatoon, Canada; 3 Instructional Design Specialist, Gwenna Moss Centre for Teaching Effectiveness, University of Saskatchewan, Saskatoon, Canada

## Correction

Following publication of the original article [1], author 2 pointed out that his name has since changed from Adiba Islam to Adiba Ashrafi.

## References

[1] John Moraros, Adiba Ashrafi, Stan Yu, Ryan Banow and Barbara Schindelka. *BMC Medicial Education* (2015) 15:27. DOI 10.1186/s12909-015-0317-2 10.1186/s12909-015-0317-2 PMC4363198 25884508

